# Hybrid optical-electronic convolutional neural networks with optimized diffractive optics for image classification

**DOI:** 10.1038/s41598-018-30619-y

**Published:** 2018-08-17

**Authors:** Julie Chang, Vincent Sitzmann, Xiong Dun, Wolfgang Heidrich, Gordon Wetzstein

**Affiliations:** 1 Bioengineering Department, Stanford University, Stanford, CA 94305 USA; 2 Electrical Engineering Department, Stanford University, Stanford, CA 94305 USA; 3 Visual Computing Center, King Abdullah University of Science and Technology, Thuwal, 23955 Saudi Arabia

## Abstract

Convolutional neural networks (CNNs) excel in a wide variety of computer vision applications, but their high performance also comes at a high computational cost. Despite efforts to increase efficiency both algorithmically and with specialized hardware, it remains difficult to deploy CNNs in embedded systems due to tight power budgets. Here we explore a complementary strategy that incorporates a layer of optical computing prior to electronic computing, improving performance on image classification tasks while adding minimal electronic computational cost or processing time. We propose a design for an optical convolutional layer based on an optimized diffractive optical element and test our design in two simulations: a learned optical correlator and an optoelectronic two-layer CNN. We demonstrate in simulation and with an optical prototype that the classification accuracies of our optical systems rival those of the analogous electronic implementations, while providing substantial savings on computational cost.

## Introduction

Deep neural networks have found success in a wide variety of applications, ranging from computer vision to natural language processing to game playing^1^. Convolutional neural networks (CNNs), capitalizing on the spatial invariance of various image properties, have been especially popular in computer vision problems such as image classification, image segmentation, and even image generation^2–4^. As performance on a breadth of tasks has improved to a remarkable level, the number of parameters and connections in these networks has grown dramatically, and the power and memory requirements to train and use these networks have increased correspondingly.

While the training phase, during which network weights are learned, is often considered the slow stage, large models also demand significant energy and storage during inference due to millions of repeated memory references and matrix multiplications. To increase efficiency, many strategies have been employed to compress CNNs while maintaining performance, including pruning, trained quantization, Huffman encoding, and altered architectural design^5,6^. On the hardware side, there are now specialized processing units for machine learning, such as IBM’s TrueNorth chip, Movidius’s vision processing units (VPUs), and Google’s tensor processing units (TPUs)^7^. Other inference-focused efforts aimed at embedded vision applications have tried to incorporate a portion of the image processing on the sensor, eliminating or reducing the need to shuttle full image data to a processor^8,9^. Computational efficiency of CNNs continues to be an area of active research, and it remains difficult for embedded systems such as mobile vision, autonomous vehicles and robots, and wireless smart sensors to deploy CNNs due to stringent constraints on power and bandwidth.

Here we explore a complementary strategy that incorporates a layer of optical computing prior to either analog or digital electronic computing, improving performance while adding minimal electronic computational cost and processing time. Optical computing is tantalizing for its high bandwidth, high interconnectivity, and inherently parallel processing, all potentially at the speed of light^10^. Certain operations can be performed in free space or on a photonic chip with little to no power consumption, e.g. a lens can take a Fourier transform “for free”^11,12^. An optimizable and scalable set of optical configurations that preserves these advantages and serves as a framework for building optical CNNs would be of interest to computer vision, robotics, machine learning, and optics communities. Initial research on optical neural networks (ONNs) was spurred by the capability of optics to perform the expensive matrix multiplication of a fully connected layer^13–16^. Recently there has been renewed interest in ONNs, both in academic research^17–20^ and in industry (Fathom Computing, Lightelligence, Optalysis). However, the ONN literature referenced does not involve convolutional layers, which have become essential in computer vision applications. In addition, these methods have been developed using coherent light as the signal, which makes them difficult to adapt into a computational camera system.

We take steps toward the goal of an optical CNN from a computational imaging perspective, integrating image acquisition with computation via co-design of optics and algorithms. Computational cameras exploit the physical propagation of light through custom optics to encode information about a scene that would be lost in a standard 2D image capture^21–25^. Here we present a computational imaging system modeled after a feed-forward CNN that assists in performing classification of input images. By pushing the first convolutional layer of a CNN into the optics, we reduce the workload of the electronic processor during inference. Furthermore, an imaging scenario where the input signal is already an optical signal easily allows for propagation through additional passive optical elements prior to sensor readout. The ASP Vision system previously explored the idea of a hybrid optical-electronic CNN, using angle sensitive pixels (ASPs) to approximate the first convolutional layer of a typical CNN, but it was limited to a fixed set of convolutional kernels^26^. A concurrent work incorporates optimizable elements into a neural-network-inspired multilayer optical system but does not attempt to create a CNN^20^. In contrast, our aim is to design a system with an optical convolutional layer optimized for a specific classification problem, thereby demonstrating low-power inference by a custom optoelectronic CNN.

In this work, we propose a design for an optical convolutional (opt-conv) layer with an optimizable phase mask that exploits the inherent convolution performed by a linear, spatially invariant imaging system. We first test our design in two simulated models for image classification. As the simplest application of a convolutional layer, we learn an optical correlator consisting of a single convolutional layer that performs template matching on images, as has been explored for optical target detection and tracking^27–30^. Next, we show how the proposed opt-conv layer could fit within a larger hybrid optoelectronic CNN, in which the output of the convolutional layer is fed into a digital fully connected layer. In both cases, we successfully demonstrate that the classification accuracy of the simulated optoelectronic configuration rivals that of an unconstrained electronic implementation of the same network structure. Finally, we validate the results of our simulations by fabricating the optimized phase mask and building a prototype of the hybrid optoelectronic two-layer network that performs classification on the grayscale CIFAR-10 dataset. Whereas a single digital fully connected layer yields around 30% classification accuracy on the test dataset, our prototype achieves over 44% accuracy, providing an improvement of almost 50% of the single layer’s performance while operating at a comparable computational cost. In contrast, for similar accuracy improvement, the addition of a standard convolutional layer would have increased the computational cost by another order of magnitude. Hence our results show how a hybrid optoelectronic convolutional neural network that includes an initial layer of optical computing can offer considerable gains in performance while minimally impacting the latency or power consumption of the system.

## Results

### Optical convolutional layer

**Figure 1:**
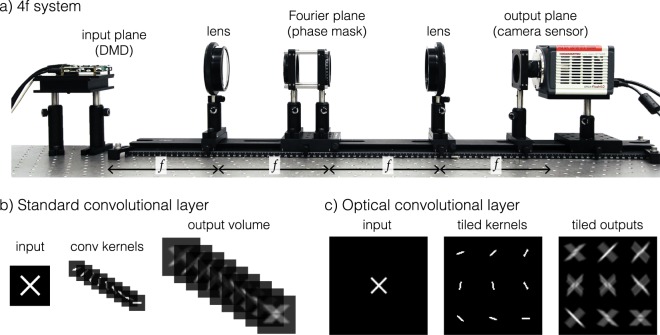
Optical convolutional layer design. (**a**) Diagram of a 4*f* system that could be adapted to implement optical convolutional (opt-conv) layers by placing a phase mask in the Fourier plane. (**b**) The standard components of a digital convolutional layer, including an input image, a stack of convolutional kernels, and a corresponding output volume. (**c**) The equivalent components in an opt-conv layer, where the kernels and outputs are tiled in a 2D array instead of stacked in the depth dimension.

A CNN typically begins with a sequence of convolutional layers, each of which performs pattern matching with a set of learnable visual filters. A standard convolutional layer takes an input volume of depth *C*_in_, performs a series of correlations with a set of *C*_out_ kernels each with depth *C*_in_, and outputs a new volume of depth *C*_out_ (Fig. 1b). The correlation of each kernel across the width and height of the input volume produces a 2D activation map, and stacking the *C*_out_ activation maps for all kernels forms the output volume of depth *C*_out_. In a CNN that takes a monochromatic image as the input, the first convolutional layer has *C*_in_ = 1. In subsequent convolutional layers, *C*_in_ may grow or shrink based on *C*_out_ of the previous layer. A tunable bias variable, ${\bf{b}}\in {{\mathbb{R}}}^{{C}_{{\rm{out}}}}$, can be added to the output, with each element *b*_*i*_ of the bias vector applied across the *i*^th^ activation map. Hyperparameters of a convolutional layer include the size of the kernel, the stride with which the kernel is applied, and the padding of the input volume. The size of the kernel refers to the height and width of the kernel in pixels, and the stride describes by how many pixels the kernel is shifted on the input volume before taking the next inner product. If we start with the left side of the kernel aligned at the left side of the image and take unit strides across the width until the right sides align, the width of the output volume will be smaller than that of the input. Padding is a way of extending the borders of the input volume to allow for an equal input and output size. Here we assume a stride of one, meaning the kernel is shifted by one pixel at a time, and sufficient zero-padding of the input such that the output volume has the same height and width as the input. For a unit-strided, zero-padded convolutional layer with *C*_out_ kernels, before adding biases, each channel *j* of the output volume can be described as:

1 $${I}_{{\rm{o}}{\rm{u}}{\rm{t}},j}=\sum _{i=1}^{{C}_{{\rm{i}}{\rm{n}}}}{I}_{{\rm{i}}{\rm{n}},i}\,\star \,{W}_{i,j}\,,{\rm{f}}{\rm{o}}{\rm{r}}\,j\in 1,2,...{C}_{{\rm{o}}{\rm{u}}{\rm{t}}},$$

where ($\star$) signifies a 2D correlation and *W*_*i*,*j*_ refers to the *i*^th^ channel of the *j*^th^ kernel.

We propose an optical implementation of the first convolutional layer in an imaging context. We assume the light in the system is spatially incoherent, which is true of most imaging situations. For the purposes of this derivation, we also assume monochromatic illumination, which can be approximated even under ambient lighting with the addition of a spectral filter. Since *C*_in_ = 1 for our first layer, we do not explicitly write the summation of Equation (1). Furthermore, since our optical signals are continuous in all spatial dimensions, we denote these quantities as functions of *x* and *y*. In linear optical systems, the image formation is often modeled as a spatially invariant convolution of the scene with the point spread function (PSF) of the system:

2 $${I}_{{\rm{out}}}(x,y)={I}_{{\rm{in}}}(x,y)\,\star \,{\rm{PSF}}(x,y),$$

where ($\star$) signifies a 2D convolution. One way to achieve this is with a “4*f* system,” a basic telescope consisting of two convex lenses, each with focal length *f*, performing a cascade of two Fourier transforms. The system is so-named due to the distance of 4*f* between the input and output image planes. The first lens is placed one focal distance *f* away from the object plane, producing a Fourier plane another distance *f* after the first lens. The second lens is then placed another distance *f* from the Fourier plane, producing an inverted conjugate image plane a total distance 4*f* from the original object plane (Fig. 1a). The Fourier plane of such a system can be modulated in amplitude and phase, akin to a bandpass filter in signal processing, which alters the PSF of the system^12^.

This optical 2D convolution can be viewed as a convolutional layer with *C*_in_ = *C*_out_ = 1 and the flipped PSF as the convolutional kernel. We will also refer to the flipped PSF as the kernel since the flipping operation can be accomplished trivially both in computation and in optics (by flipping the phase mask). Now suppose we want *C*_out_ = *n*, where $n > 1$. By spatially tiling the multiple kernels as the PSF of the system in an *A* × *B* grid, the output becomes the convolution of the input image with multiple 2D kernels, but now the *n* outputs are tiled laterally (Fig. 1c) instead of stacked in depth (Fig. 1b). Consideration can be taken to ensure these outputs are non-overlapping by adjusting the shifts Δ*x* and Δ*y*, if desired. The PSF can be described as

3 $${\rm{PSF}}(x,y)=\sum _{a=1}^{A}\sum _{b=1}^{B}{W}_{aB+b}(x,y)\,\star \,\delta (x-a{\rm{\Delta }}x+{\rm{\Gamma }},y-b{\rm{\Delta }}y+{\rm{\Gamma }}),$$

where Γ is an offset that can be added to shift the PSF so that it is centered at the origin of the optical axis. The resulting image formation is described by

4 $${I}_{{\rm{out}}}(x,y)=[{I}_{{\rm{in}}}\,\star \,{\rm{PSF}}](x,y)=\sum _{a=1}^{A}\sum _{b=1}^{B}[{I}_{{\rm{in}}}\,\star \,{W}_{aB+b}](x,y)\,\star \,\delta (x-a{\rm{\Delta }}x+{\rm{\Gamma }},y-b{\rm{\Delta }}y+{\rm{\Gamma }}),$$

where *W*_*i*_ corresponds to the *i*^th^ convolutional kernel for a single channel input image.

Thus we have a way to convolve a single input image with multiple 2D kernels, where the multiple output channels are tiled across the 2D image plane instead of stacked in a third depth dimension. In extensions where multiple input channels are desired, the input could be tiled in a similar way, allowing for the tiled output of the previous layer to be processed and fed into a second opt-conv layer. This would result in a slighly altered opt-conv behavior where convolutional kernels are cycled among the inputs, the effect of which would be interesting to explore in future work. This opt-conv layer can be optimized in a similar manner as a standard convolutional layer using stochastic gradient descent (SGD) methods. After discretizing the 2D space, the desired shape and number of kernels can be initialized and tiled into a larger single-channel convolutional layer, which can then be optimized for a specific imaging task. However, there exists a major constraint: the incoherent PSF we work with cannot have any negative values, as a negative light intensity is not physically realizable. We consider this constraint in more detail in the simulations.

#### Phase mask optimization

After PSF optimization, the task still remains of finding an optical element that can produce the desired PSF. The Fourier plane of a 4*f* system can be modulated with an aperture transfer function (ATF) to control the incoherent PSF of the system:

5 $${\rm{PSF}}(x,y)=| {\mathcal F} \,\{{\rm{ATF}}({k}_{x},{k}_{y})\}(x,y){|}^{2},$$

where ${\mathcal F}$ signifies a 2D Fourier transform, ${k}_{x}=\frac{x}{\lambda f}$ and ${k}_{y}=\frac{y}{\lambda f}$ denote spatial frequencies, *λ* is the wavelength of light, and *f* is the focal length of the lenses. The ATF is a complex function that can be decomposed into amplitude and phase as

6 $${\rm{ATF}}({k}_{x},{k}_{y})=A({k}_{x},{k}_{y})\cdot {e}^{i{\rm{\Delta }}\varphi ({k}_{x},{k}_{y})},$$

where local amplitude *A* can be implemented with a transparency mask and local phase shifts Δ*ϕ* can be realized with a diffractive optical element (DOE) of spatially varying thickness. To prevent loss of light and reduce the fabrication complexity of the ATF-defining optical element, we keep the amplitude constant, *A* = 1, and restrict our optimization to phase only. To find a phase profile that can generate the desired discretized convolutional kernel, PSF_opt_, our problem becomes:

7 $$\mathop{{\rm{m}}{\rm{i}}{\rm{n}}{\rm{i}}{\rm{m}}{\rm{i}}{\rm{z}}{\rm{e}}}\limits_{{\rm{\Phi }}}\parallel {{\rm{P}}{\rm{S}}{\rm{F}}}_{{\rm{o}}{\rm{p}}{\rm{t}}}-|{\mathscr{F}}\{{e}^{i{\rm{\Delta }}{\rm{\Phi }}}\}{|}^{2}{\parallel }_{{\rm{F}}}^{2},$$

where $\parallel \cdot {\parallel }_{{\rm{F}}}$ denotes the Frobenius norm and Φ is the discretized 2D phase profile matrix of the DOE to be optimized. We continue to use an SGD-based learning approach for this optimization problem. More details are given in the Methods section.

### Simulated results

#### Learned optical correlator

**Figure 2:**
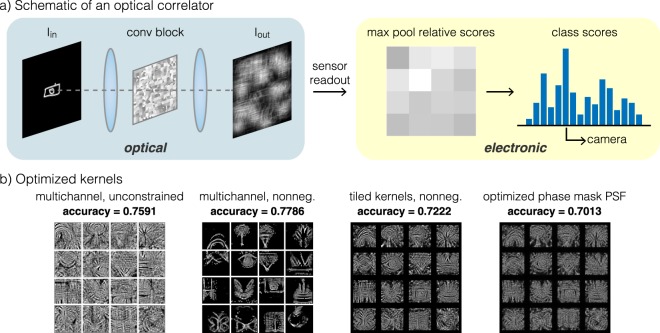
Learned optical correlator. (**a**) Schematic of an optical correlator, where the conv block consists of the 4*f* system shown in Fig. 1. (**b**) Characteristic optimized kernels of a multichannel unconstrained digital convolutional layer, a multichannel nonnegative digital convolutional layer, a single channel opt-conv layer with tiled kernels, and the PSF produced by phase mask optimization with the previous optimized tiled kernels as the target.

To confirm that our proposed opt-conv layer would function as expected in the simplest possible setup, we simulate a classification system with a single convolutional layer. The schematic of this classifier is shown in Fig. 2a. An input image from one of *N* classes is projected into the opt-conv block with an optimizable PSF. The output image of the convolutional layer is partitioned into an array of *N* sub-images corresponding to the *N* classes, and then a score for each class is calculated by taking the maximum intensity pixel within each sub-image. The predicted class of the input image is the class with the highest score.

For this set of simulations, our dataset consists of images from the Google QuickDraw dataset, *N* = 16. We choose this dataset because it is more complex than the MNIST handwritten digit dataset (for which over 90% accuracy can be achieved with a single layer; see Supplementary Fig. S1) but still relatively manageable for an optical correlator. We compare several different versions of a single convolutional layer network, beginning with the benchmark of a standard multichannel convolutional layer with unconstrained weights. Next we add in nonnegative constraints to the weights, which is necessary since the intensity of light must be nonnegative. From here we move to an optically plausible 2D convolution with only a single channel, where we tile the smaller kernels within the area of a large kernel. In all cases, we initialize 16 convolutional kernels of size 32 × 32, which are stacked for the multichannel digital convolution and tiled for the optical correlator. We do not use any bias variables in the single-layer network.

We show representative optimized kernels for each of these models in Fig. 2b. Visual inspection shows that all cases learn an average shape of the objects in the various QuickDraw categories, such as a rainbow in the top left and a butterfly on the bottom left. We list all categories in the Methods section. Averaging over five trials, we find that the stacked multichannel cases achieve accuracies of 75.9% with unconstrained weights and 77.9% with nonnegative weights, and this discrepancy lies within the variation of individual experiments (see Supplementary Table S1 for standard deviations). The tiled kernel configuration has slightly decreased performance but is still able to learn reasonable filters that achieve 72.2% accuracy. Our results show that for a single convolutional layer, the nonnegative constraint does not detrimentally impact classification accuracy. Next we select a set of tiled kernels as a target PSF for phase mask optimization. The final image in Fig. 2b shows the PSF produced by the optimized phase mask, which closely matches the target PSF shown to its left. More important than visual similarity though is the functionality of the optimized phase mask, which we assess with the same Google QuickDraw image classification task. Using a simulated optical convolution with this phase mask, the classification model achieves a test accuracy of 70.1%, comparable to the accuracy of digital convolution with the target tiled kernels. These results suggest that the opt-conv layer is able to learn behavior similar to a digital convolutional layer, and that our phase mask optimization procedure is able to find phase mask templates that produce the desired PSFs.

#### Hybrid optoelectronic CNN

Next we evaluate whether an opt-conv layer can also be integrated into a hybrid optoelectronic CNN, which would allow it to be used in more difficult classification tasks than can be handled by an optical correlator. In this set of simulations we add in two more fundamental components of a CNN: a nonlinear activation layer and a fully connected layer. Inclusion of the nonlinear activation layer expands the hypothesis space of the network beyond that of a linear classifier (Supplementary Fig. S2), and the additional fully connected layer provides more parameters to increase the model’s expressive power. In particular, we insert the popular rectified linear unit (ReLU) nonlinearity between the opt-conv and fully connected layers, which applies the unit ramp function to each element of the input^1^, in effect preserving only positive activations.

**Figure 3:**
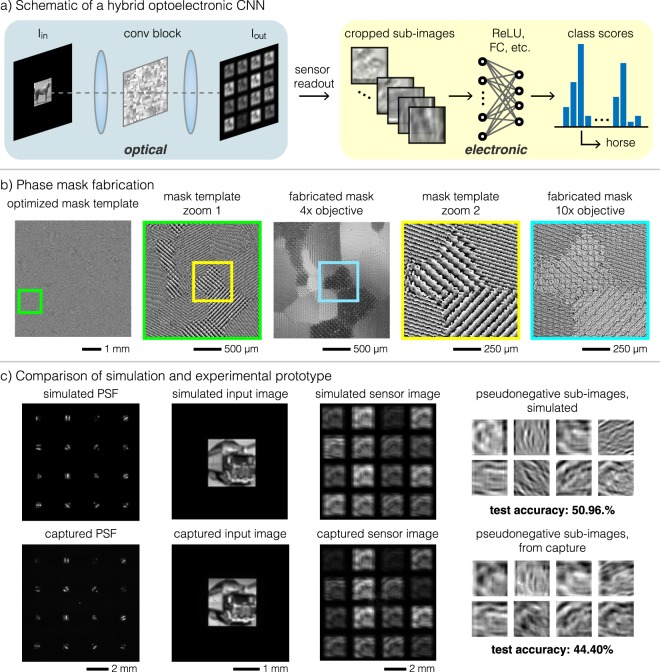
Hybrid optoelectronic CNN. (**a**) Schematic of a model with a single opt-conv layer, after which the sensor image is processed and fed into subsequent digital CNN layers. (**b**) The optimized phase mask template and microscope images of the fabricated phase mask, at different zoom levels. (**c**) Comparison of simulated and captured versions of the PSF produced by the phase mask, a sample input image, the respective sensor image, and pseudonegative sub-images after subtraction of corresponding positive (top two rows) and negative (bottom two rows) sub-images.

In the hybrid model, we again begin with a single opt-conv layer, but now we additionally process the sensor image into a stack of sub-images and feed the data as input to the electronic portion of the network (Fig. 3a). We refer to this network as a two-layer CNN, since activation layers and other layers without tunable weights are not typically counted when calculating the depth of a neural network. In a standard electronic two-layer CNN, the convolutional layer would likely be the more computationally expensive layer (Table 1). The substitution of the digital convolutional layer with an opt-conv layer allows the convolutions to be performed without any electronic computation while still maintaining flexibility in the first layer weights. To more clearly demonstrate the performance gains with and without the opt-conv layer, we apply this model to a more complex problem: classification of grayscale CIFAR-10 images.

**Table 1:** Hybrid optoelectronic CNN performance for grayscale CIFAR-10 classification with various models. Classification accuracies of simulated models are the average of five trials. Standard deviations (std. dev.) are computed for the simulated models. Learned parameters and FLOPs are split into those for the optical and electronic parts of the network, when relevant.

Method	Test accuracy	Learned parameters	FLOPs
	mean ± std. dev.	optical/electronic	optical/electronic
**Simulation:**			
fully connected (FC) only	29.8 ± 0.5%	−/10,250	−/20,480
digital conv > ReLU > FC, unconstrained	51.9 ± 1.3%	−/82,586	−/1,490,954
digital conv > ReLU > FC, nonnegative	36.3 ± 0.5%	−/82,586	−/1,490,954
digital conv > ReLU > FC, pseudonegative	51.8 ± 0.6%	−/83,234	−/2,818,058
optical conv > ReLU > FC, pseudonegative	51.0 ± 1.4%	104,976/81,938	3,779,136/180,234
**Physical experiment:**			
optical conv > ReLU > FC, pseudonegative	44.4%	−/81,938	−/180,234

The benchmark for this network architecture is an unconstrained CNN with a standard multichannel convolutional layer (9 × 9 filters, *C*_out_ = 8), normalization, a ReLU nonlinearity, and a fully connected layer with *N* = 10 output scores. We now include tunable bias variables for the convolutional and fully connected layers, both of which are applied in the electronic portion of the network. When the nonnegative constraint is applied to the kernel weights and input images are also nonnegative, the standard ReLU nonlinearity essentially becomes an identity function, as the ReLU no longer receives any negative inputs. To ensure that the output of the convolutional layer still spans the nonlinear region, we normalize and shift the sensor readout to zero-mean before feeding into the ReLU layer, similar to batch normalization in deep learning terminology^31^. For consistency, we do this both in the unconstrained and constrained models.

The unconstrained two-layer benchmark CNN achieves a classification accuracy of 51.9% on the test dataset. While the nonnegative constraint did not detrimentally impact the optical correlator, we found that once the additional fully connected layer was added in this model, nonnegative constraints resulted in a substantially worse performance of 36.3% (Table 1) due to a severely limited hypothesis space. To address this issue, we propose a *pseudonegative* variant where half of the kernels were labeled positive and the other half negative, requiring a digital subtraction of the corresponding positive and negative sub-images after sensor readout, as shown by Fahrat *et al*.^13^. While this requires an additional computational step, it considerably improved performance to match the unconstrained weight case, bringing simulated test accuracy back to 51.8%.

After learning a set of pseudonegative kernels, we prepare the PSF for phase mask optimization by tiling the kernels across a single large kernel, spacing them sufficiently so that the corresponding output sub-images do not overlap. A sample optimized phase mask for the tiled pseudonegative kernels, its corresponding PSF, and an example input-output image pair are shown in Fig. 3b,c. Because the resultant phase mask may not produce the exact same target kernel, we run an additional fine-tuning optimization of the bias weights and fully connected layer weights to ensure that they store information relevant to the new PSF. After fine-tuning, we simulate inference with the optoelectronic CNN by cropping the output of the opt-conv layer into sixteen sub-images, stacking in the depth dimension, resizing to the original dimension if necessary, and feeding into the rest of the model. The resizing step is necessary if the sensor outputs need to be downsampled to standard CIFAR-10 dimensions. The test results shown in Table 1 indicate that the simulated optical convolution is able to perform nearly as well as a digital convolution.

In Table 1, we also include a count of the number of learned parameters and floating point operations (FLOPs) required during a single inference task to highlight the potential savings when using an optically-addressed convolutional layer. Learned parameters include the convolutional kernel weights, fully connected layer weights, biases, and phase mask profiles. The FLOP count includes the standard floating point operations of additions and multiplications (see Supplementary Information for more details). Table 1 also lists counts of learned parameters and FLOPs for the optical portion of the simulated hybrid model; these are the parameters and operations that are stored in the optical element and automatically carried out by light propagation in the experimental prototype. Convolutional layers, especially with larger kernel sizes, are computationally expensive due to the repeated multiplications and additions while sliding the kernel over the entire input image. In contrast, optical convolutions occur naturally as light propagates through the 4*f* system, and PSF or kernel size does not impact system latency. In our two-layer CNN, addition of the digital convolutional layer improves classification accuracy from 29.8% to 51.9%, but at the cost of over one million additional FLOPs. In the simulated optical convolution, the accuracy improves comparably to 51.0%, but only 180,234 FLOPs are required in the electronic portion of this model, approximately 12% of the 1,490,954 FLOPs required by the fully digital implementation. As an additional benefit, by effectively storing the weights of the convolutional kernels in the optical element, we can reduce the memory requirements of the electronic part of the system. We are able to fully achieve these savings in our physical prototype, which we now discuss.

### Experimental results

We fabricated the optimized phase mask from the hybrid ONN simulations as a DOE and built a prototype of the opt-conv layer (see Methods). We use a fabricated DOE as opposed to an active spatial light modulator (SLM) to minimize power consumption during inference. The phase mask feature size was set at 6 μm, allowing for comfortable alignment during fabrication and a large aperture for light efficiency. The fabrication process successfully reproduced the desired pattern, based on comparison of microscope images of the fabricated mask and corresponding areas of the mask template. Furthermore, the experimental PSF, captured by projecting a small point through the system, appears qualitatively very similar to the target PSF (Fig. 3c), though there is a zeroth-order artifact in the captured PSF due to imperfect diffraction efficiency of the physical phase mask. To calibrate projection of grayscale CIFAR-10 images through the optical system, we adjusted the projected image size so that the ratio of the input image width to the kernel width matched that in the simulation (32:9), and we aligned the sub-images to fall on a 2D Cartesian grid on the sensor. Alternatively, for a fixed object size, instead of the 1:1 symmetric 4*f* system that we built, an asymmetric 4*f* system consisting of two lenses of different focal lengths could be employed to adjust the field of view of the system. Again, simulated sensor images closely match the captured sensor image (Fig. 3c). After cropping out sub-images, resizing to original CIFAR-10 pixel dimensions, and subtracting the eight negative kernels from the eight positive kernels, we start to see some differences between the simulation and experiment, possibly due to slight errors in alignment, imperfections of the PSF, and irregularities of the digital micromirror device (DMD) used to project test images. Nonetheless, the characteristic edge filtering effect of a first convolutional layer is still evident.

We captured the full CIFAR-10 dataset with our prototype and shuttled the data to a GPU for the electronic portion of the system. As in the hybrid ONN simulations, we use the training dataset to fine-tune the downstream parameters of the network, feeding in the captured images paired with their known labels. After fine-tuning, we achieve a 46.9% accuracy on the validation set and a 44.4% accuracy on the test set. The addition of the opt-conv layer yields over 14% accuracy improvement over the single fully connected layer, without incurring the computational cost of a digital convolutional layer (Table 1). The prototype test accuracy falls slightly below the simulated opt-conv test accuracy of 51.0%, likely due to hardware imperfections unaccounted for in simulation. Since the actual phase mask had only 16 discrete heights instead of the continuous heights allowed in the phase mask optimization, the PSF produced by the physical phase mask, though very similar, did not exactly match the target PSF. In addition, the boundaries of the convolved sub-images may not have precisely aligned with the sensor pixels due to the different pixel sizes of the DMD and camera sensor, resulting in alignment imperfections when subtracting kernels to yield the pseudonegative sub-images. The zeroth-order artifact produced an unwanted copy of the image that overlapped with the nearest sub-image regions, which may have compromised some of the information stored in the sub-images. On the display side, there were a few dead pixels on the DMD, which can be seen by examining the differences between the simulated input image and the actual projected version, captured by imaging without a phase mask (Fig. 3c). All of these differences may have contributed to the small gap between the simulated and physical hybrid ONN models.

## Discussion

In this work, the overarching question we explore is how custom optimized optics can be used to absorb a portion of the computational cost during inference by a CNN. We choose the task of image classification as a representative CNN application and design a computational camera system that uses a combination of optical elements and digital computation to automatically classify a scene. Specifically, we propose an opt-conv layer that imitates a standard convolutional layer, operates with zero power consumption, and can be optimized during a training phase in a standard deep learning framework. Our goal throughout this work was not to match the highest accuracy achieved with a state-of-the-art ultra-deep CNN, but rather to understand the behavior of the opt-conv layer and assess its potential in a hybrid optoelectronic CNN. Hence we set up simple models in which individual layer contributions would be more obvious than in larger models, including a single layer optical correlator and a two-layer CNN. In both simulation and physical experiment, we find that our opt-conv layer successfully learns the same type of behavior as a digital convolutional layer in analogous networks, achieving comparable performance during inference tasks without the extra computational cost of the digital implementation. We were able to circumvent the physical constraint of nonnegative weights in an incoherent PSF through a pseudonegative model, where we included a digital subtraction of half of the output image from the other half. In the future, it will be interesting to explore other creative solutions, perhaps exploiting polarization or using multiple datastreams.

Several areas of improvement can be investigated to mature our optical prototype into a compact and portable imaging system. For usage in-the-wild, this system should be able to capture incoherent images of the surrounding environment and process them through the optimized pipeline. Our prototype already operates with spatially incoherent light, but our models assumed monochromatic, i.e. temporally coherent, illumination and grayscale datasets, allowing us to disregard chromatic aberrations in proof-of-concept experiments. In a natural environment, a monochromatic image could be achieved through a narrow bandpass filter, but important color information would be lost. In the Supplementary Information, we discuss how color-sensitive kernels could be optically realized and include a supporting simulation showing improved performance (Supplementary Fig. S3). Alternatively, chromatic aberrations could be harnessed to extract color information^32^, and incorporation of hyperspectral imaging techniques could further enhance the information captured by the device^33^. Secondly, our prototype uses standard bulk optics and free-space propagation in a tabletop 4*f* system, achieving reduced power consumption at the cost of increased system size and weight. More compact configurations can be explored, perhaps by optimizing multiple flat DOEs to replace the larger lenses^20^, integrating the phase mask into an embedded vision system, or exploring the potential of flat optics and metasurfaces^34,35^. Photonic integrated circuits could alternatively be used to miniaturize the design onto a chip and allow for the training phase to occur directly on the optics^17,18,36,37^.

CNNs and their applications appear to be spreading through all aspects of our lives, from self-driving cars to face recognition to medical diagnostics. The complex models require previously unfathomable power demands, making certain usages impractical or impossible, especially in embedded systems. Efforts to reduce complexity and increase speed while maintaining performance are being sought both in software and hardware. Incorporation of optical computing would be complementary to most existing approaches, relieving both the software and electronic hardware of a portion of the computation. Optical computing has potential for lower latency than electronic computation, which could be highly valuable in interactive systems such as autonomous vehicles where fast decision-making is crucial. Additionally, optical implementations have the potential to expand beyond traditional operations of CNNs, perhaps by harnessing wave optics and quantum optics in new ways. In this article, we highlight the benefits of the paradigm of pushing part of the computation into optics, particularly when the initial signal is received in the form of light, by demonstrating substantial gains in classification accuracy without the computational cost of the analogous digital layer. Our work thus offers new and valuable insights into the potential of optical CNNs in the future of high-efficiency autonomous computer vision systems.

## Methods

### Datasets

The dataset used in the optical correlator simulation was comprised of drawings from the Google QuickDraw dataset. We used 28 × 28 grayscale bitmaps downloaded directly from the publically available datasets (github.com/googlecreativelab/quickdraw-dataset) in 16 categories: apple, book, bowtie, butterfly, cake, camera, cat, chair, crown, diamond, eye, fish, hand, ice cream, lollipop, and rainbow. The training dataset consisted of the first 8,000 images in each of the 16 categories, and the testing set the next 100 images in each category. The hybrid ONN simulation used the standard CIFAR-10 dataset^38^. For grayscale images, we took the mean over the original three color channels.

### Network and training details

We simulated our models in a TensorFlow framework, using built-in functions whenever possible. Since some of the convolutional kernels and PSFs we tried to optimize were much larger than those of a standard CNN, we used an FFT-based convolution to increase computation speed when possible. All training was run on an Nvidia Titan X (Pascal) GPU. The various optimizations we ran can be divided into two groups: PSF optimization and phase mask optimization. PSF optimization includes all the models that only consider the kernels and PSFs—not the optical elements that produce them—of the classification model. In these models, the input is an image from the dataset, and the output is a set of scores corresponding to the possible categories. During training of these classification models, we also feed in the true category labels and employ a standard cross-entropy loss, from which we backpropagate gradients and update weights with the ADAM optimizer for 10,000 iterations (learning rate = 5e-4). In some cases, we required convolutional kernel weights to be nonnegative. To impose this constraint, we included an element-wise squaring operation on the initial weight variables, effectively requiring them all to be nonnegative.

Phase mask optimization refers to the models where we simulate image formation through the opt-conv layer consisting of a 4*f* system with a phase mask in the Fourier plane. Here the tensor to optimize is the height profile of the phase mask, from which the system PSF is computed. The height profile is iteratively updated until it produces a PSF that matches a target, typically one produced from the previously described PSF optimization. This model takes sample images and a target PSF as input and computes both a ground truth where the input image is convolved with a target PSF and an actual output where the input image is propagated through the current phase mask. The objective is to minimize the ${\ell }_{2}$ loss between the ground truth and model output. We run the ADAM optimizer for 20,000 (correlator) or 50,000 (two-layer CNN) iterations, with a learning rate decaying from 5e-4 to 1e-9.

The optimized phase mask may not produce the exact same PSF as the target PSF. This is important for the two-layer optoelectronic CNN model, as the stored weights of the downstream fully connected layer are not guaranteed to still be relevant. Hence, we also ran a fine-tuning optimization where we fixed the phase mask produced by the phase mask optimization, then allowed the model to re-optimize the weights of the fully connected layer given the corresponding PSF. For the refinement optimization, we re-ran the classification model optimizer for 5000 iterations (ADAM, learning rate = 5e-4).

### Phase mask fabrication

The DOE used in the hybrid CNN prototype was fabricated via multi-level photolithography^39^. The optimized phase mask design was discretized to 16 height levels, which could then be realized by cycling through four rounds of photolithography and Reactive Ion Etching (RIE) processes. For a transmission substrate, we used a 0.5 mm thick Fused Silica wafer (4 inch diameter). In each fabrication cycle, a 100 nm Cr layer was first deposited on the wafer, followed by a spin-coating of 0.6 μm photoresist layer (AZ1505) and soft-bake at 100 °C for 60 seconds. The pattern template was transferred from a photomask to the photoresist under 15 dose ultraviolet (UV) light exposure, and the exposed areas of the photoresist layer were removed in a developer wash (MIL AZ726) for 20 seconds. Subsequently, the exposed areas of the Cr layer area were removed in Cr etchant for 90 seconds, which transferred the pattern to the Cr layer. In the RIE step, the material (SiO_2_) in the open areas was removed by a mixture of Argon and SF_6_ plasma, which transferred the pattern from Cr layer to the wafer.

Each fabrication cycle doubles the possible number of microstructure levels from the previous profile. By repeating this cycle four total times with different amounts of etching, 16 levels of microstructures were created on the wafer, with a maximum etching depth of 1125 nm for all 16 levels. Finally, to block stray light outside the mask area, an opaque 200 nm Cr layer was deposited on the entire surface and then etched to only allow light to pass through the active phase mask area. The fabricated phase mask was then cut and mounted in the optical setup described next.

### Optical setup

In our hybrid ONN experiment, we projected CIFAR-10 grayscale images from a digital micromirror device (DMD) through a custom opt-conv layer, capturing and feeding the intermediate images to the electronic fully connected layer. The images were projected on the pixels of a DMD (Texas Instruments DLP LightCrafter), illuminated by 532 nm laser light (Coherent Inc.) made spatially incoherent by scrambling through two rotating diffusers (1500 and 600 grit, Thorlabs)^40^. Images were resized on the DMD such that the relative size of the input image and the convolutional kernels would match that of the simulated model used during training. An encoding gamma (*γ* = 0.5) was also applied to the image intensities before sending to the DMD to account for the inherent decoding gamma of the display. Displayed DMD images were relayed through a 4*f* system consisting of two convex *f* = 200 mm lenses (Edmund Optics), with the fabricated phase mask placed at the center of the Fourier plane. Output images were captured by an ORCA-Flash 4.0 sCMOS camera (Hamamatsu). The exposure time was set at 385 ms to take advantage of the full well capacity of the sensor pixels, consequently decreasing the impact of Poisson noise. This exposure time could have been decreased, as long as it allowed for sufficient rotation of the diffusers to impart spatial coherence, but we were not prioritizing capture speed during our experiment. The captured images were then shuttled to an Nvidia Titan X (Pascal) GPU for downstream processing. See Supplementary Fig. S4 for images of the complete setup.

## Electronic supplementary material

Supplementary Information

## Data Availability

Source code and captured datasets are available at https://github.com/computational-imaging/opticalCNN.
