# Supplementary material for: Hybrid optical-electronic convolutional neural networks with optimized diffractive optics for image classification
Source: Sci Rep. 2018 Aug 17;8:12324. doi: 10.1038/s41598-018-30619-y (PMC6098044; doi:10.1038/s41598-018-30619-y)
Supplement: Supplementary file 1 — Supplementary Information [file 41598_2018_30619_MOESM1_ESM.pdf]

Supplementary Information: Hybrid optical-electronic convolutional neural networks with optimized diffractive optics for image classification

Julie Chang[1,*], Vincent Sitzmann[2], Xiong Dun[3], Wolfgang Heidrich[3], and Gordon Wetzstein[2,*]

[1]Bioengineering Department, Stanford University, Stanford, CA 94305, USA
[2]Electrical Engineering Department, Stanford University, Stanford, CA 94305, USA
[3]Visual Computing Center, King Abdullah University of Science and Technology, Thuwal 23955, Saudi Arabia
[*]{jchang10, gordon.wetzstein}@stanford.edu

This document provides additional information and figures to support the paper "Hybrid optical-electronic convolutional neural networks with optimized diffractive optics for image classification."

1 Single layer simulations on MNIST dataset

Image classification of the MNIST dataset of handwritten digits[1] is a basic task that can be used to confirm whether a classification model operates as expected. We compared two classification models on this task: a single fully connected layer and a single channel convolutional layer with the same number of trainable weights that could potentially be achieved with an opt-conv layer. In the fully connected layer model, the scores for the N classes were read directly from the output vector, $\mathbf{y} \in \mathbb{R}^N$. In the convolutional model, the output image was partitioned into equally sized sub-images, and the N scores were given by the maximum intensity of a pixel within the sub-image. For convenience, we chose to only use digits one through nine, $N = 9$, so that the output image could be divided into a 3×3 array of square regions. We define the models in a TensorFlow framework and use a cross-entropy loss and the ADAM optimizer to learn the weights.

Given this scoring approach, we hypothesized that the convolutional model would learn an average representation of each of the N classes in approximately the corresponding regions of the large convolutional kernel, similar to how a single fully connected layer learns a template of each class. We see that this is indeed the case in Supplementary Fig. S1, which shows the optimized weights for both cases. The test accuracy for the convolutional model was slightly higher than the fully connected layer, which was expected since a convolutional template matching allows more flexibility in the exact positioning of the input image. These results motivated us to develop the opt-conv layer in more detail. We also noticed that both accuracies were already over 90%, which did not give much room to improve. This led us to use more difficult datasets in the main paper.

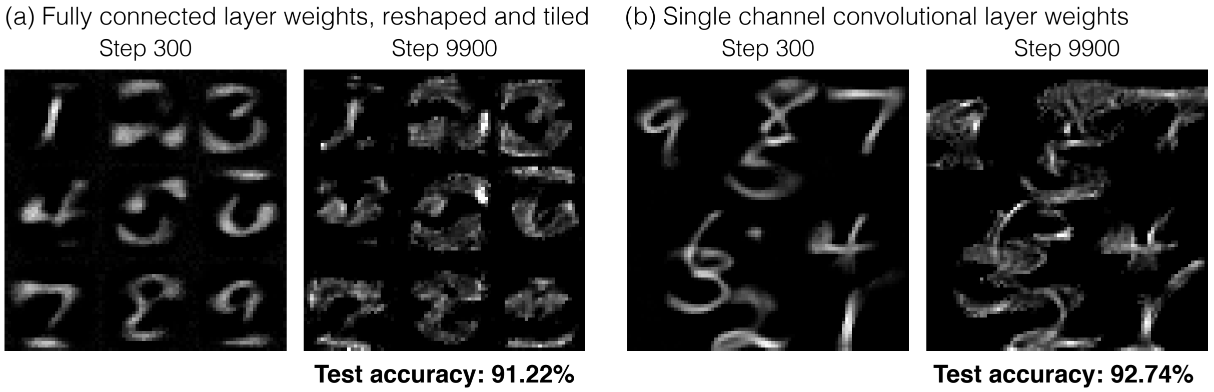

Figure S1. MNIST (1-9) classification with single layer models. Visualization of the weights after 300 and 9900 steps in (a) the fully connected model and (b) the single channel convolutional model. The weights of the fully connected layer are reshaped into the shape of the input image and tiled in numerical order.

2 Understanding the constraints of an optical neural network

Compared to a vanilla neural network, an optical neural network (ONN) imposes several constraints that are important to understand when attempting to design an ONN. To provide some intuition, we created a 2D toy classification problem with a fully connected network (no convolutional layers) for visualization of the effects of these constraints. A fully connected layer typically includes a matrix multiplication and a vector addition, called a bias, allowing for an affine transformation between the input and output. In an ONN that does not inject or subtract fixed values of light at each layer, this bias variable cannot be addressed. Another major constraint of computing with light intensity as the signal is that light intensity cannot be negative. The multiplicative weights of the fully connected layer therefore must be nonnegative, unless we switch to computing with coherent light. With both the no-bias and nonnegative constraints in place, all the values in the model are nonnegative and fall into the linear region of the standard rectified linear unit (ReLU) layer, essentially making the model a constrained linear classifier. Inspired by batch normalization, which normalizes the output of a layer to be zero-mean and unit-variance to prevent saturating nonlinearities[2], we introduced a "shifted ReLU" to potentially recover the benefits of the nonlinear activation layers. This nonlinearity has an extra parameter that allows the threshold of the ReLU to shift away from zero:

$$x_{\text{out}} = \max(\delta, x_{\text{in}}), \tag{1}$$

where δ is a shift parameter that can also be learned by the model. Such an activation layer may not necessarily be optically realizable, but we include it to maintain nonlinear capabilities in the ONN.

We generated four different datasets, shown at the top of each of the four columns of Supplementary Fig. S2, and used variants of a fully connected neural network to classify these datasets. All of the models had the same network architecture:

$$\text{input} \in \mathbb{R}^2 \Rightarrow \text{FC}(32) \Rightarrow \text{FC}(64) \Rightarrow \text{FC}(128) \Rightarrow \text{FC}(64) \Rightarrow \text{FC}(2) \Rightarrow \text{scores} \in \mathbb{R}^2,$$

where $\text{FC}(n)$ signifies a fully connected layer with output dimension of n. We now describe the variations on this model in order from top to botton of Supplementary Fig. S2.

The first two models are included as two points of reference of standard classification models. In the linear model, no nonlinear activation layers were placed between the fully connected layers, resulting in linear decision boundaries for all the datasets. Without nonlinear activation layers, the expressivity of the model would remain the same regardless of the number of fully connected layers, as all of them could be collapsed into a single layer. For the "vanilla FC" model, we included a ReLU nonlinearity between all of the consecutive fully connected layers. This allowed the model to learn nonlinear decision boundaries, which were especially important in second two datasets. No additional constraints are imposed on these two models.

We add constraints in the following three models. The effect of imposing a no-bias constraint is that all decision boundaries now intersect the origin, since a zero input maps to a zero output. In this model, the standard ReLU nonlinearity was also added, but the model did not seem as flexible in creating nonlinear decision boundaries. Next we impose the nonnegative constraint, still allowing for a bias and the standard ReLU nonlinearities. The effect here is that the slope of the decision boundary, as it is depicted in our visualizations, cannot be positive. This is apparent in the second dataset, which is easily classified by the unconstrained linear classifier, but is unable to be properly separated by the nonnegative model. In the final variation, we impose both the nonnegative and no bias constraints and modify the ReLU to a shifted ReLU. We see the benefit of including a nonlinearity most clearly in the fourth dataset. The decision boundary curves partly between the two categories as best as it can without including any segment of positive slope.

These examples depict how the no-bias and nonnegative constraints can drastically reduce the hypothesis space of an ONN. The shifted ReLU was valuable in regaining some nonlinear behavior, but the full hypothesis space of the vanilla FC was not recovered. While image-based data is very different from these 2D datasets, similar effects likely apply. For these reasons, we took an optoelectronic approach to the optical CNN, allowing for computation-saving benefits from the first opt-conv layer while maintaining the relative flexibility of electronic layers.

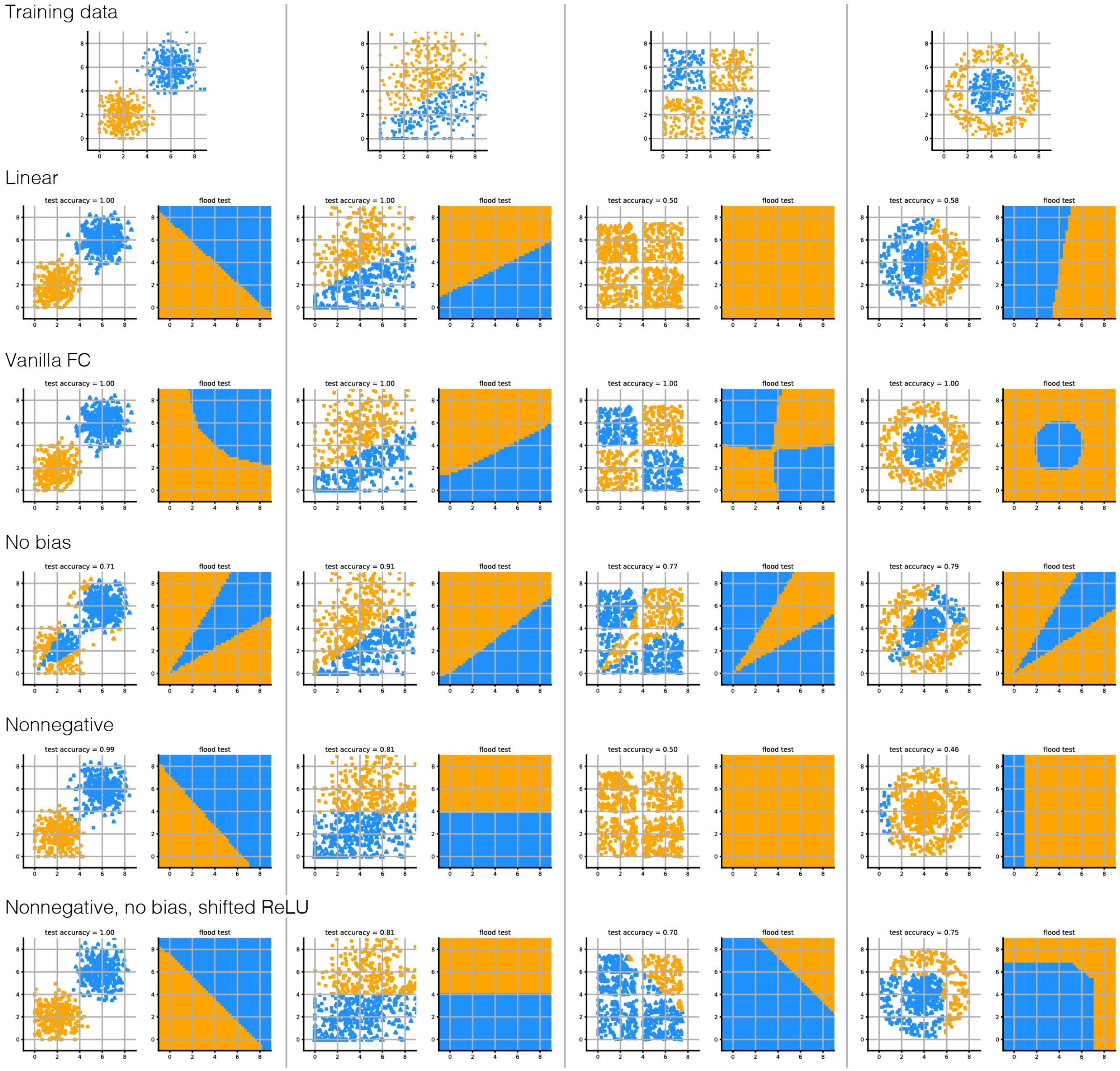

Figure S2. 2D classification with constrained models. Test classification results when fully connected neural networks with various constraints are trained to classify four different training datasets. The colors in the training data graphs show the ground truth labels of the data. The colors in the model-specific graphs show the predicted classes, while the shapes of the markers indicate the correct classes (triangle corresponds to blue in the training data, square corresponds to orange in the training data). The flood test graphs allow for clearer visualization of the learned decision boundaries.

3 Optimized convolutional kernels of the two-layer optoelectronic CNN

In Supplementary Fig. S3, we show representative kernels learned in the two-layer optoelectronic CNN to more clearly illustrate the pseudonegative scheme discussed in the main paper. In the unconstrained case, the model directly learns edge-detecting kernels oriented at different angles. When the nonnegative constraint is imposed, the optimized kernels look noticeably different. The original behavior is lost, and the performance suffers significantly (Supplementary Table S2). To circumvent this, we introduce pseudonegative kernels, which doubles the number of kernels but recovers the performance of the unconstrained case. Eight kernels are designated as positive and another eight as negative, but all 16 still have the nonnegative constraint imposed. After image capture, the sub-images of corresponding positive and negative kernels are subtracted, effectively allowing for negative values in the kernels. The subtraction of the eight positive and eight negative pseudonegative kernels is shown in the top right, and the result, as desired, looks very similar to the unconstrained kernels. Based on the successful simulated digital convolution results, we proceeded with the pseudonegative kernels for phase mask optimization.

We also explored the potential gains when using colored filters in our opt-conv layer. Removing the assumption of monochromatic illumination or narrow bandpass filtering, spectral filters could be tiled over the sensor to effectively separate the tiled kernels into color channels. To train this version of the CNN, we used the original color RGB CIFAR-10 images. In simulation, the incorporation of color information did result in higher classification accuracy of 57.6%, compared to grayscale image classification accuracy of 51.0% (Supplementary Table S2). These preliminary results are promising and would be interesting to explore in future work. Phase mask optimization for the color-sensitive kernels would require additional attention to chromatic aberrations in a diffractive optical element, which has previously been addressed by Peng et al.[3]

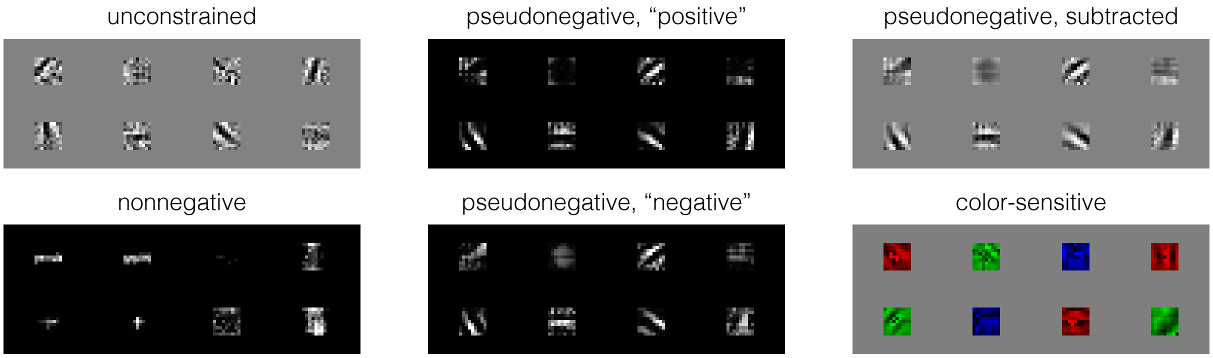

Figure S3. Optimized convolutional kernels of the two-layer CNN. In the first column are the unconstrained and nonnegative kernels. The second column shows the pseudonegative kernels designated positive and negative. In the third column are the subtracted pseudonegative kernels and an example of color-sensitive kernel optimization.

4 Extended simulation results

We include a summary of the results from the optical correlator simulations in Supplementary Table S1, also displayed in Fig. 2 of the main paper. The simulated optical convolution with the optimized phase mask has a fixed PSF, so only one test trial was run. In addition, in Supplementary Table S2 we show extended results from the two-layer hybrid optoelectronic CNN. We bold the lines that were not included in Table 1 of the main paper. The unrefined version of the “optical conv” simulation refers to immediate use of the optimized phase mask without fine-tuning the downstream electronic weights.

Table S1. Classification accuracies of learned optical correlators.

Method	Test accuracy (mean $\pm$ std. dev.)
standard multichannel convolution, unconstrained	75.9 $\pm$ 2.3%
standard multichannel convolution, nonnegative	77.9 $\pm$ 0.7%
tiled-kernel, single-channel convolution, nonnegative	72.2 $\pm$ 1.3%
simulated optical convolution with optimized phase mask	70.1%

Table S2. Hybrid optoelectronic CNN performance for CIFAR-10 classification with various models. Classification accuracies of simulated models are the average and standard deviation of five trials. Learned parameters and FLOPs are split into those for the optical and electronic parts of the network, when relevant. Datasets are grayscale except in the "optical conv (color)" simulation. Results that were not included in Table 1 of the main paper are bolded.

Method	Test acc.	Learned parameters<br>optical / electronic	FLOPs<br>optical / electronic
**Simulation:**			
FC only	$29.8 \pm 0.5\%$	- / 10,250	- / 20,480
**digital conv only, unconstrained**	**$35.9 \pm 1.0\%$**	**- / 820**	**- / 1,658,880**
**digital conv only, nonnegative**	**$27.5 \pm 1.6\%$**	**- / 820**	**- / 1,658,880**
digital conv > ReLU > FC, unconstrained	$51.9 \pm 1.3\%$	- / 82,586	- / 1,490,954
digital conv > ReLU > FC, nonnegative	$36.3 \pm 0.5\%$	- / 82,586	- / 1,490,954
digital conv > ReLU > FC, pseudonegative	$51.8 \pm 0.6\%$	- / 83,234	- / 2,818,058
**optical conv > ReLU > FC, pseudonegative (unrefined)**	**$41.4 \pm 3.8\%$**	**104,976 / 81,938**	**3,779,136 / 180,234**
optical conv > ReLU > FC, pseudonegative **(fine-tuned)**	$51.0 \pm 1.4\%$	104,976 / 81,938	3,779,136 / 180,234
**digital conv (color) > ReLU > FC, pseudonegative**	**$57.6 \pm 0.6\%$**	**- / 83,234**	**- / 2,818,058**
**Physical experiment:**			
optical conv > ReLU > FC, pseudonegative	44.4%	- / 81,938	- / 180,234

5 FLOP calculations

In Supplementary Table S2, we included a comparison of the number of FLOPs required for a single inference task using each of the network configurations. By convention, FLOP counts tally floating point additions and multiplications. ReLU, pooling, and reshaping operations are not included.

Fully connected layer. The operations of a fully connected layer consist of a matrix multiply of the weight matrix and the vectorized input followed by a vector addition with the bias vector. The number of FLOPs in a fully connected layer is defined by the input dimension d_{in} and output dimension d_{out}:

$$d_{\text{out}}(2d_{\text{in}} - 1) + d_{\text{out}} \tag{2}$$

The corresponding number of learned parameters is $d_{\text{in}} \cdot d_{\text{out}} + d_{\text{out}}$. As an example, the "FC only" simulation has an input size of 32×32 and an output size of 10. The number of learned parameters is calculated by $32 \cdot 32 \cdot 10 + 10 = 10,250$. The FLOP count is calculated by $10(2 \cdot 32 \cdot 32 - 1) + 10 = 20,480$.

Convolutional layer. In a convolutional layer, each output pixel comes from the dot product of a convolutional kernel and a region of the input image or volume. In this work, all of our convolutional layers have a single layer input image, stride of 1, and zero-padding such that the output is the same size as the input. For a convolutional layer with k kernels, each of field size $f\times f$, and an input image with width w, the FLOP count is calculated by:

$$k(w^2(2f^2 - 1) + w^2) \tag{3}$$

The total number of trainable parameters in this convolutional layer, including biases, is $k(f^2 + 1)$. As an example, the "digital conv only, unconstrained" simulation has $k = 10$ kernels with field size $f = 9$ and input width of $w = 32$. This corresponds to $10(9^2 + 1) = 820$ parameters and $10(32^2(2 \cdot 9^2 - 1) + 32^2) = 1,658,880$ FLOPs.

Simulated optical convolution. To simulate the optical convolution, we use an FFT-based convolution. We approximate the FLOP count of a 2D FFT on an image of size $N\times N$ as $N^2 \log N^2$. The additional learned parameters of the opt-conv layer store the height profile of the phase mask. The simulated optical convolution includes one forward FFT, one element-wise multiplication with the optical transfer function (OTF) defined by the phase mask, and one inverse FFT. The learned parameter and FLOP counts are affected by the resolution of the phase mask and the extra padding placed around the convolutional kernels. In the physical experiment, all of these operations are performed by the optics, so only the fully connected layer contributed to the counts.

6 Optical prototype

Supplementary Fig. S4 shows a top and side view of the optical prototype described in the Methods of the main paper. The top view shows the illumination path toward the digital micromirror device (DMD) and the following imaging path through the optical convolutional block to the camera sensor. The side view more clearly shows the components of the optical convolutional block. Inset into the top view is an image of the fabricated phase mask illuminated from above with white light.

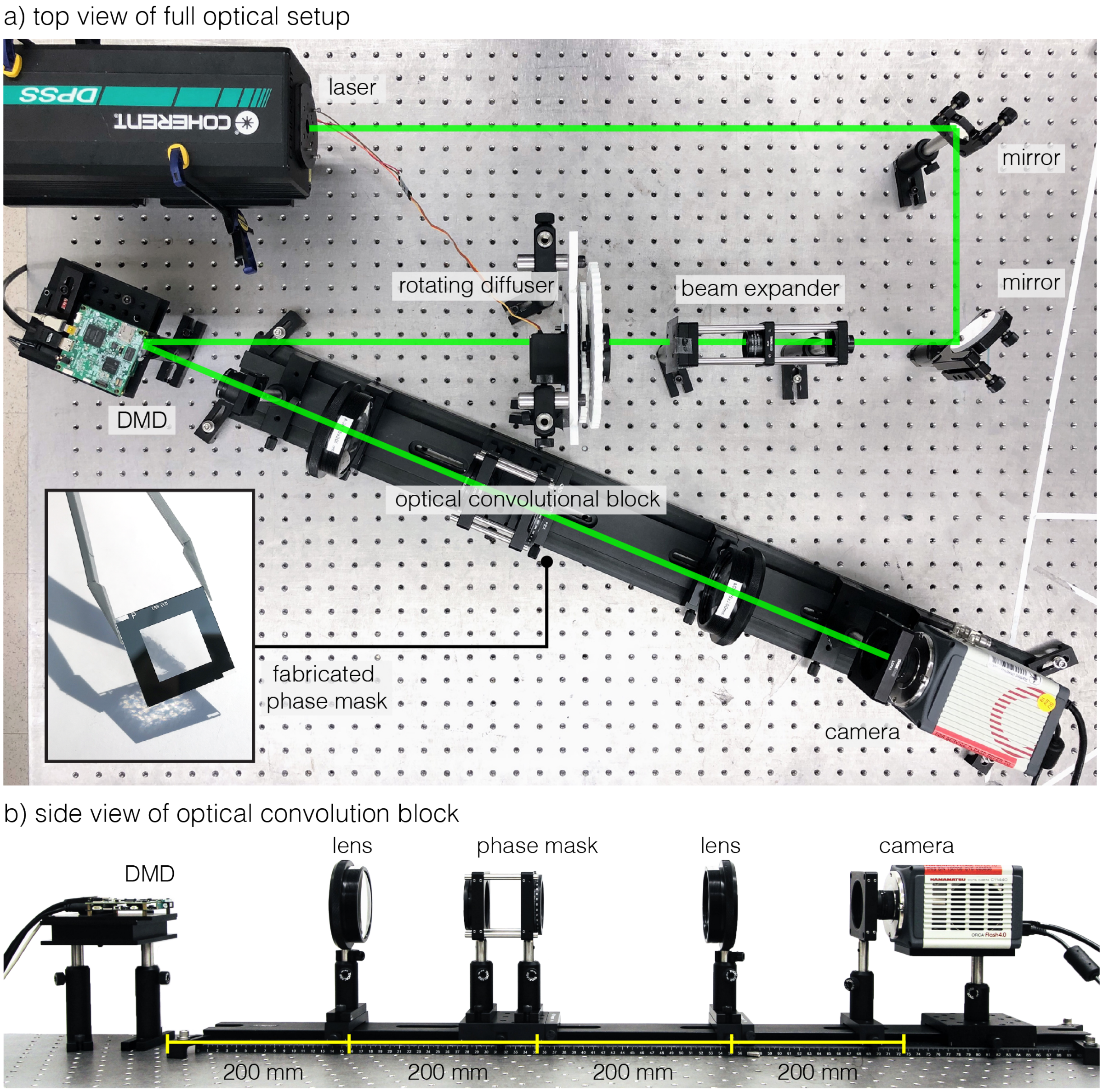

Figure S4. Optical prototype. (a) Top view of entire optical path and (b) side view of the optical convolutional block.

References

1. LeCun, Y., Cortes, C. & Burges, C. The MNIST database of handwritten digits. http://yann.lecun.com/exdb/mnist (2012).
2. Ioffe, S. & Szegedy, C. Batch normalization: Accelerating deep network training by reducing internal covariate shift. *arXiv preprint arXiv:1502.03167* (2015).
3. Peng, Y., Fu, Q., Heide, F., & Heidrich, W., The diffractive achromat: full spectrum computational imaging with diffractive optics, in *ACM SIGGRAPH* (2016).
